# Diabetes Medication Use and Blood Lactate Level among Participants with Type 2 Diabetes: The Atherosclerosis Risk in Communities Carotid MRI Study

**DOI:** 10.1371/journal.pone.0051237

**Published:** 2012-12-26

**Authors:** Morgana L. Mongraw-Chaffin, Kunihiro Matsushita, Frederick L. Brancati, Brad C. Astor, Josef Coresh, Stephen O. Crawford, Maria Inês Schmidt, Ron C. Hoogeveen, Christie M. Ballantyne, Jeffery Hunter Young

**Affiliations:** 1 Department of Epidemiology, Johns Hopkins Bloomberg School of Public Health, Baltimore, Maryland, United States of America; 2 Department of Medicine, The Johns Hopkins Medical Institutions, Baltimore, Maryland, United States of America; 3 Department of Medicine, University of Wisconsin School of Medicine and Public Health, Madison, Wisconsin, United States of America; 4 Davita Clinical Research, Inc, Minneapolis, Minnesota, United States of America; 5 School of Medicine, Federal University of Rio Grande do Sul, Porto Alegre, Brazil; 6 Department of Epidemiology, School of Public Health at Chapel Hill, University of North Carolina, Chapel Hill, North Carolina, United States of America; 7 Section of Atherosclerosis and Vascular Medicine, Department of Medicine, Baylor College of Medicine, Houston, Texas, United States of America; 8 Center for Cardiovascular Disease Prevention, Methodist DeBakey Heart Center, Houston, Texas, United States of America; College of Tropical Agriculture and Human Resources, University of Hawaii, United States of America

## Abstract

**Background:**

The objective of this study is to compare lactate levels between users and non-users of diabetes medications under the hypothesis that the level of lactate is a marker of oxidative capacity.

**Methods:**

The cross-sectional data of 493 participants aged 61–84 with type 2 diabetes who participated in the Atherosclerosis Risk in Communities Carotid MRI study were analyzed using survey weighted linear regression.

**Results:**

Median plasma lactate level was 8.58 (95% CI: 8.23, 8.87) mg/dl. Comparing users of diabetic medications with non-users, thiazolidinedione use was significantly associated with lower lactate level (7.57 (6.95–8.25) mg/dL vs. 8.78 (8.43–9.14) mg/dL), metformin use with a slightly higher lactate level (9.02 (8.51–9.58) mg/dL vs. 8.36 (7.96–8.77) mg/dL), and sulfonylurea and insulin use were not associated with lactate level. After adjustment for demographic and lifestyle factors, the plasma lactate level for thiazolidinedione users was 15.78% lower than that for non-users (p<0.001). Considering use of each medication separately and in combination did not change the results.

**Conclusion:**

In conclusion, thiazolidinedione use was associated with lower plasma lactate level compared to non-use and metformin use was only marginally associated with a slightly higher lactate level. These results are consistent with the previously demonstrated effects of diabetes medications on oxidative metabolism. Further investigation of the role that diabetes medications play in improvement of oxidative metabolism is warranted.

## Introduction

Oxidative capacity is the balance between the body's demand for energy and the ability to provide that energy through oxidative pathways. The use of lactate as a marker of systemic imbalance in oxygen demand and availability allows for the relatively new emergence of population based studies on oxidative capacity [1]–[3]. Decreased oxidative capacity, or mitochondrial dysfunction, is associated with increased insulin resistance, although the causal direction of this association is still in question [4]–[7]. Recent work in the Atherosclerosis Risk in Communities(ARIC) Carotid MRI study used higher blood lactate as an indicator of reduced oxidative capacity to investigate this association [1].

The relationship between oxidative capacity and insulin resistance leads to the hypothesis that interventions that lower insulin resistance may also impact oxidative capacity. The relationship of the biguanides with lactate level has been well-studied due to the clinical concern about lactic acidosis. While a recent Cochrane review suggests that patients taking metformin do not have higher lactate levels, only a few studies with small sample sizes were available and an increase in lactate level would be consistent with some of metformin's physiologic effects [8]. Specifically, lactate is the primary precursor for gluconeogenesis. Since metformin decreases hepatic gluconeogenesis, it decreases lactate utilization and potentially increases lactate levels [9].

In contrast, the direct effect of other diabetes medications on lactate levels is largely unknown. Thiazolidinediones (TZDs), for example, may influence lactate levels through prevention of gluconeogenesis in the liver like metformin, as well as through increases in oxidative phosphorylation in skeletal muscle and adipose tissue [9], [10]. TZDs are also known to increase oxygen availability more generally by decreasing adipocyte size and increasing vasodilatation [9], [10]. Furthermore, TZDs significantly increased exercise capacity as measured by Vo_2max_ in a clinical trial [11]. These effects on increased oxidative capacity in both adipose tissue and skeletal muscle may result in an overall decrease in plasma lactate levels. Insulin may also increase oxidative capacity through stimulation of mitochondrial biogenesis and increased vasodilatation [6], [12]–[15]. Based on the differing effects of medication classes on oxidative pathways, each class of diabetes medication should have a different association with lactate levels. To test these hypotheses, we investigated the difference in plasma lactate levels between users and non-users of diabetes medications among ARIC Carotid MRI Study participants.

## Materials and Methods

### Study population

The original ARIC study has been described in detail elsewhere [16]. This study used a subset of the ARIC Carotid MRI ancillary study, which included 2066 of the original ARIC participants, oversampled for high carotid intima-media thickness (IMT) [1], [17]. The primary goal of the ARIC CAR-MRI ancillary study was to identify factors related to carotid atherosclerosis measured with carotid MRI. Given the expense and inconvenience of MRI assessment, the number of ARIC participants required was minimized by oversampling patients with high IMT levels, thereby generating a sufficient range of IMT levels for analyses. Since the Carotid MRI study oversampled participants with high IMT, our sample is over represented by these participants. Given the known sampling probability ARIC Carotid-MRI study participants, however, we can adjust all traditional analyses (such as linear regression in this case) to reflect the distribution of factors in the entire ARIC cohort, with a measure of uncertainty as reflected in the confidence intervals. Therefore, we incorporated inverse probability weights in all analyses to compensate for the sampling design and provide unbiased estimates that reflect the entire ARIC population. This subset was chosen as plasma lactate was available for these participants through an ancillary study. Lactate measurements were not available for the rest of the cohort. Participants missing fasting plasma lactate levels or other variables of interest as well as those with a body mass index less than 18.5 kg/m^2^ were excluded from the lactate ancillary study subset (n = 182). Only participants with type 2 diabetes, determined at the Carotid MRI visit by a fasting blood glucose higher than 126 mg/dL, previous diagnosis, or treatment, were included (n = 496). Finally, those missing data on diabetes medication use were excluded (n = 3). The final sample size for this analysis includes 493 participants aged 61 to 84.

### Ethics Statement

The study was approved by the institutional review board of the Johns Hopkins Bloomberg School of Public Health (IRB No: H.34.04.08.16.A1) and all participants provided full written consent.

### Exposure

Diabetes medication use was defined as use of any diabetes medication in the four weeks prior to the Carotid MRI study clinic visit and is based on review of medication containers brought to the study visit by participants. Similarly, use of each specific class of diabetes medications (TZDs, metformin, insulin, and sulfonylurea) was defined as any use of that specific medication type in the previous four weeks assessed by container review. For the primary analysis, participants could be taking more than one type of diabetes medication and the comparison group is participants not taking that medication type.

### Outcome

Measurement and validation of plasma lactate levels in the ARIC Carotid MRI Study has been described previously [1]. Briefly, plasma lactate concentrations were measured on a Roche Hitachi 911 auto-analyzer using an enzymatic reaction whereby lactate was converted to pyruvate and hydrogen peroxide, which was then measured through the generation of dye by the reaction of hydrogen peroxide and peroxidase. Reliability of repeated plasma lactate measurements in this sample was high (reliability coefficient = 0.93) [1].

### Covariates

Potential confounding variables of interest included age, sex, race, education level (completed high school or not), body mass index (BMI), waist to hip ratio, leisure-time physical activity using the Baecke questionnaire [18], smoking, alcohol consumption, markers of kidney function (creatinine and glomerular filtration rate) and insulin resistance (triglyceride/HDL ratio). Demographic variables were taken collected at baseline (ARIC visit 1). All other data were collected during the Carotid MRI Study visit.

### Statistical analysis

Due to the ARIC Carotid MRI sampling strategy, the basic characteristics of the study sample were reported as stratified sample-weighted means and linearized standard errors by use of each diabetes medication. Plasma lactate level was log transformed to account for deviations from the normal distribution and to improve model fit. Lactate level was reported as geometric means and 95% confidence intervals and differences in log lactate level were reported as back transformed percent differences and 95% confidence intervals. Multivariable analysis was performed using linear regression on cross-sectional data using stratified sample weights that account for the sampling scheme in the Carotid MRI Study. These weights include consideration for oversampling of intima-media thickness by study site and effectively remove the influence of oversampling for high IMT [1]. Results from linear regression on log lactate level were also reported as back transformed percent differences and 95% confidence intervals. Models were adjusted first for demographic factors (age, sex, race, and education) as well as physical activity level and body mass index. Primary models were checked for sensitivity to smoking and alcohol consumption. Additional sensitivity analyses were conducted to determine whether substituting waist to hip ratio for body mass index, adjusting for markers of kidney function such as creatinine and glomerular filtration rate, or adjusting for overlapping medication use influenced the results. All analyses were performed using STATA 11 [19].

## Results

**Figure 1:**
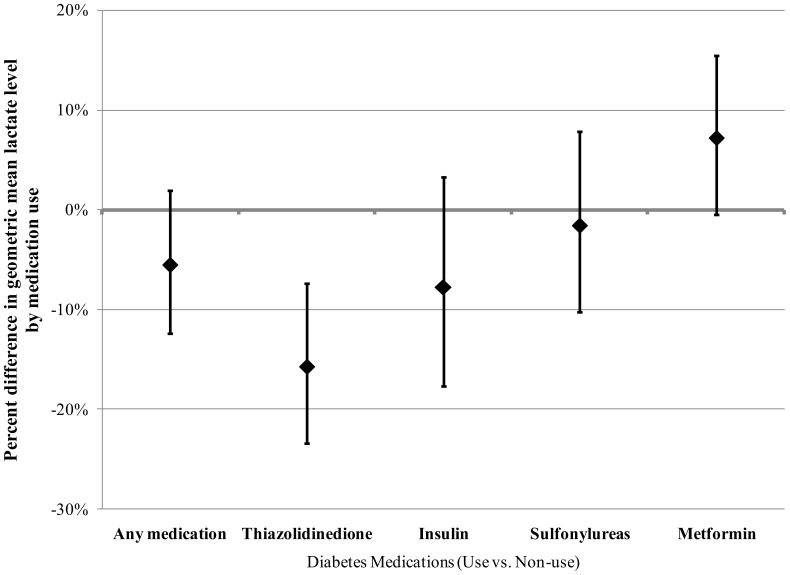
Adjusted percent differences in plasma lactate in the ARIC Carotid MRI Study. Adjusted differences in plasma lactate (vs non-users of diabetes medications) in users of thiazolidinediones, Insulin, Sulfonylureas, and Metformin in those with type 2 diabetes in the ARIC Carotid MRI Study: Percent differences and 95% confidence intervals. Adjusted for age, sex, race, and education, sport and leisure activity, and body mass index.

**Figure 2:**
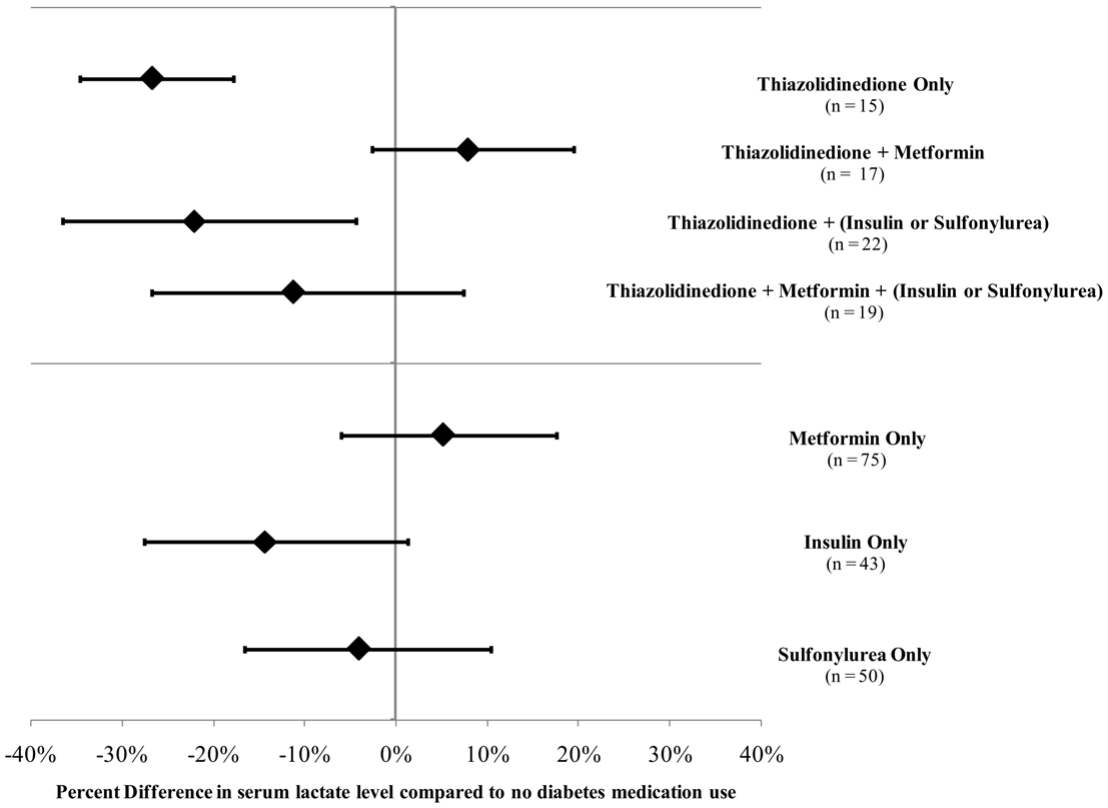
Effect of medication combinations on percent differences and 95% confidence intervals in serum lactate level. Effect of medication combinations on percent differences and 95% confidence intervals in serum lactate level (mg/dl) among persons with type 2 diabetes in the ARIC Carotid MRI Study. All comparisons based on a model containing age, sex, race, education, sport and leisure activity, and body mass index. Diabetes medication use was constructed as a factor variable with mutually exclusive groups representing each medication separately and in combination. All groups are compared to no diabetes medication use. Not all combinations are shown.

Overall median plasma lactate level in the study sample was 8.58 (95% CI: 8.23, 8.87) mg/dl compared to 7.13 (95% CI: 7.00, 7.27) mg/dl in the non-diabetic cohort. Participant characteristics by diabetes medication use are displayed in Table 1. In unadjusted analyses, metformin use was marginally associated with higher mean plasma lactate level (percent difference = 8.0%, *P* = 0.05) and thiazolidinedione (TZD) use was associated with lower lactate levels (percent difference = −13.8%, *P* = 0.002), but other diabetes medication use was not associated with differences in lactate (Table 1). The results were similar after simultaneous adjustment for age, sex, race, education level, physical activity, and body mass index (Figure 1). Results were also similar when waist to hip ratio was used instead of body mass index (data not shown) and after further adjustment for smoking status, alcohol consumption, markers of kidney function (creatinine and glomerular filtration rate) and insulin resistance (triglyceride/HDL ratio), and pre-existing co-morbidities (stroke, coronary heart disease, and heart failure) (Table 2). Adjustment for other diabetes medication use also produced similar results (data not shown). In analyses restricted to participants taking only one type of diabetes medication compared to those taking no diabetes medication, the association between TZD use and lower lactate levels was more evident (Figure 2). Among those who used both metformin and TZDs, the association of TZD use with lactate level was attenuated.

**Table 1:** Characteristics of 493 Adults Aged 61–84 With Type 2 Diabetes in the ARIC Carotid MRI Study by Diabetes Medication Use.

	Any Diabetes Medication Use		Thiazolidinedione Use		Metformin Use		Insulin Use		Sulfonylurea Use	
Characteristic	No	Yes	No	Yes	No	Yes	No	Yes	No	Yes
**N**	169	324	420	73	329	164	411	82	370	123
**Female (%)**	54.2 (4.82)	50.0 (3.52)	51.1 (3.09)	50.6 (7.39)	53.0 (3.48)	47.2 (4.90)	49.4 (3.13)	60.2 (6.76)	**54.4 (3.27)**	**41.0 (5.77)**
**African American (%)**	27.3 (3.90)	31.0 (2.83)	30.5 (2.37)	23.8 (5.95)	29.6 (2.72)	29.3 (4.09)	**24.9 (2.27)**	**55.6 (6.88)**	31.1 (2.55)	24.8 (4.52)
**Age (years)**	70.7 (0.50)	70.4 (0.35)	**70.8 (0.32)**	**69.1 (0.62)**	70.8 (0.37)	70.0 (0.47)	70.4 (0.31)	71.2 (0.75)	70.5 (0.33)	70.8 (0.61)
**Completed high school (%)**	79.6 (3.61)	82.6 (2.38)	80.4 (2.20)	85.6 (4.66)	81.3 (2.41)	80.9 (3.59)	82.2 (2.13)	75.6 (5.66)	80.4 (2.38)	83.4 (3.66)
**Current drinker (%)**	40.6 (4.89)	37.4 (3.45)	36.7 (3.03)	47.2 (7.38)	39.2 (3.48)	36.6 (4.81)	40.0 (3.09)	29.1 (6.51)	38.5 (3.25)	37.8 (5.66)
**Current smoker (%)**	4.33 (2.00)	8.39 (1.98)	6.55 (1.55)	9.84 (4.52)	5.62 (1.56)	9.74 (3.07)	6.89 (1.64)	7.98 (3.38)	6.61 (1.69)	8.35 (3.12)
**BMI (kg/m^2^)**	31.1 (0.55)	31.6 (0.41)	31.2 (0.35)	32.9 (0.91)	31.3 (0.41)	31.8 (0.55)	31.3 (0.35)	32.2 (0.90)	31.7 (0.39)	30.7 (0.61)
**Waist Circumference (cm)**	108.7 (1.34)	109.5 (0.99)	108.8 (0.85)	111.7 (2.26)	108.4 (1.00)	110.8 (1.31)	108.9 (0.87)	111.0 (2.07)	109.8 (0.96)	107.5 (1.37)
**Leisure activity score**	2.25 (0.06)	2.25 (0.04)	2.24 (0.04)	2.29 (0.08)	2.22 (0.04)	2.31 (0.05)	2.27 (0.04)	2.13 (0.07)	2.24 (0.04)	2.28 (0.06)
**Sport activity score**	2.55 (0.09)	2.59 (0.06)	2.60 (0.05)	2.49 (0.11)	2.58 (0.06)	2.57 (0.08)	2.59 (0.05)	2.53 (0.11)	2.54 (0.05)	2.69 (0.09)
**Glomerular Filtration Rate**	77.7 (1.84)	75.1 (1.56)	76.1 (1.29)	74.5 (3.30)	75.1 (1.46)	77.3 (2.11)	76.6 (1.27)	72.0 (3.51)	76.2 (1.35)	74.8 (2.60)
**Creatinine (mg/dL)**	**81.8 (5.01)**	**101.3 (4.03)**	97.3 (3.63)	83.9 (6.91)	91.5 (3.84)	102.2 (5.90)	**91.8 (3.52)**	**113.9 (8.23)**	94.7 (3.66)	96.5 (6.99)
**Triglyceride/HDL ratio**	4.37 (0.29)	4.32 (0.21)	4.40 (0.19)	4.06 (0.31)	4.36 (0.21)	4.32 (0.28)	**4.49 (0.19)**	**3.50 (0.27)**	4.25 (0.17)	4.63 (0.43)
**Fasting Glucose (mg/dl)**	137.3 (2.72)	138.4 (2.85)	139.0 (2.28)	129.6 (4.62)	138.6 (2.49)	135.6 (3.67)	**134.2 (1.87)**	**156.4 (8.21)**	139.5 (2.44)	131.7 (3.77)
**HbA1c (%)**	**6.30 (0.12)**	**6.77 (0.09)**	6.57 (0.07)	6.69 (0.24)	**6.49 (0.09)**	**6.78 (0.11)**	**6.41 (0.06)**	**7.62 (0.28)**	6.56 (0.08)	6.68 (0.12)
**Lactate (mg/dl)** a	8.87 (8.38, 9.38)	8.41 (8.01, 8.83)	**8.78 (8.43, 9.14)**	**7.57 (6.95, 8.25)**	**8.36 (7.96, 8.77)**	**9.02 (8.51, 9.58)**	8.68 (8.34, 9.03)	8.07 (7.26, 8.98)	8.63 (8.28, 9.00)	8.43 (7.77, 9.16)

Bolded values indicate p-values<0.05 for the t-test of the difference for Yes – No.

All estimates are survey weighted mean (SE) except where indicated as % (SE). SE = linearized standard error.

a . Geometric mean and 95% confidence interval for blood lactate (mg/dl).

**Table 2:** Effect of Adjustment on Percent Differences and 95% Confidence Intervals in Serum Lactate Level (mg/dl) Among Persons with Type 2 Diabetes in the ARIC Carotid MRI Study.

	Any Diabetes Medication Use	Thiazolidinedione	Metformin	Insulin	Sulfonylurea
Model 1	−5.2 (−12.0, 2.2)	−13.8 (−21.7, −5.0)	8.0 (0.1, 16.5)	−6.9 (−16.8, 4.0)	−2.3 (−10.9, 7.1)
Model 2	−5.5 (−12.4, 1.9)	−15.8 (−23.4, −7.3)	7.2 (−0.4, 15.4)	−7.8 (−17.7, 3.3)	−1.6 (−10.3, 7.9)
Model 3	−3.9 (−10.3, 2.9)	−13.0 (−20.2, −5.2)	9.3 (2.0, 17.2)	−4.8 (−13.9, 5.2)	−2.0 (−10.1, 6.9)
Model 4	−2.7 (−9.4, 4.4)	−12.1 (−19.7, −3.8)	10.4 (2.7, 18.6)	−4.1 (−13.1, 5.9)	−2.0 (−10.3, 6..9)
Model 5	−2.7 (−9.3, 4.5)	−12.1 (−19.7, −3.7)	10.4 (2.8, 18.7)	−4.3 (−13.4, 5.8)	−2.2 (−10.4, 6.8)
Model 6	NA	−12.7 (−19.9, −4.7)	11.7 (3.64, 20.5)	−5.4 (−14.7, 4.8)	−2.2 (−10.7, 7.0)

Model 1 = drug use.

Model 2 = Model 1 + age + sex + race + education + physical activity +body mass index.

Model 3 = Model 2 + smoking status + alcohol consumption + creatinine + glomerular filtration rate + triglyceride/HDL ratio.

Model 4 = Model 3 + prevalent heart failure.

Model 5 = Model 4 + prevalent stroke + prevalent CHD.

Model 6 = Model 5 + mutual adjustment for other diabetes medication use (Thiazolidinediones, Metformin, Insulin, and Sulfonylurea).

All estimates survey weighted.

## Discussion

In this cross-sectional analysis, TZD use was associated with lower levels of plasma lactate, while metformin use was associated with marginally higher levels. Although insulin use was associated with marginally lower lactate level, this association was not statistically significant. Sulfonylurea use was not associated with a difference in blood lactate level.

When oxidative pathways are limited, either through insufficient oxygen availability or mitochondrial dysfunction, lactate production increases due to ongoing anaerobic glycolysis. Clinically, plasma lactate is routinely used to assess poor tissue oxygenation associated with conditions such as ischemic bowel and circulatory collapse. In the presence of these disorders, plasma lactate commonly rises to values >36 mg/dl. For 95.7% of ARIC CAR-MRI participants, however, blood lactate level was within the normal range (4.5–19.8 mg/dl) [1], [20].

The association of metformin with marginally higher lactate levels is consistent with the effects of metformin on glucose metabolism and with a large meta-analysis by Salpeter et al. [8] Salpeter et al. showed that when compared to those not taking metformin, metformin users had a slightly higher level of lactate that was borderline statistically significant. Metformin's impact on blood glucose levels is primarily through a reduction in hepatic gluconeogenesis. Since lactate is the primary gluconeogenic precursor, decreased gluconeogenesis would result in higher plasma lactate levels, assuming lactate production is unchanged.

In contrast to metformin, TZDs are associated with lower lactate levels, which may be due to their impact on oxygen availability in adipose tissue. One of TZD's major effects is to increase the number of small adipocytes, which are less insulin resistant than more mature fat cells [10]. Furthermore, their interiors are less hypoxic since oxygen can more easily diffuse across the shorter distance from cell surface to the cell's center [21], [22]. Since adipose tissue is a major source of lactate in insulin resistance and obesity [23], [24], TZD's impact on the proliferation of small, insulin-sensitive adipocytes may explain a major portion of their impact on lactate levels. Furthermore, TZDs tend to increase adipose tissue blood flow though vasodilatation, further increasing oxygen availability.

TZDs may also impact resting oxygen utilization and lactate production in skeletal muscle. As with adipose tissue, TZDs increase oxygen availability in muscle though vasodilatation. Furthermore, Regensteiner et al have shown that TZD use is associated with increased exercise capacity in individuals with type 2 diabetes, suggesting that impairments in exercise capacity seen with type 2 diabetes may be due to mitochondrial dysfunction [11]. They further suggest that the improvements in exercise capacity with TZD use may be due to TZD's effects on oxidative capacity. This study supports these findings through the demonstration of lower resting plasma lactate in patients taking TZDs. Given that TZDs exhibit multiple mechanisms that could result in lower lactate levels, the association of TZD use with lower lactate levels is consistent with the expected overall effect of TZDs.

This study has some limitations. The cross-sectional design limits investigation of temporality. Repeated measurements of plasma lactate and longitudinal data on medication use were not available for this analysis. Despite adjustment for markers of kidney function and insulin resistance, these associations may be confounded by factors related to the indication of the medications assessed, since this study did not adjust for the duration since diagnosis of diabetes or severity of diabetes progression. Moreover, direct measurements of oxygen availability, mitochondrial capacity, and demand for oxidative phosphorylation were also unavailable, requiring this study to rely on lactate as a marker of the mismatch between supply of ATP through oxidative phosphorylation and demand. Despite using plasma collected at rest and adjusting for BMI, smoking, and heart failure, we were unable to determine whether oxygen availability or mitochondrial capacity were primarily responsible for lactate variation. Additionally, it was not possible to investigate the association of lactate level with specific diabetes medications within a medication class separately. Since different TZDs have been shown to have slightly different mechanisms and side effects, it would be useful to know if the association with lactate level is consistent across different TZDs.

The strengths of this study include a community-based sample of older adults with type 2 diabetes who had measured plasma lactate levels and confirmed medication use. This study also has the advantage of being able to adjust for a large number of covariates. The lactate measurements used in the ARIC Carotid MRI study have been previously reported to have high reliability [1].

The association between TZD use and lower lactate level in this study reinforces the connection between insulin resistance and oxidative processes. While the clinical significance of a lower lactate level within the normal range is unknown, this study provides cross-sectional but population-based support for the idea that TZDs may increase oxidative capacity [11]. Furthermore, TZDs ability to improve insulin sensitivity may be enhanced by their impact on oxidative utilization. The association of TZD use with lower lactate level supports the interest in developing new PPAR-gamma agonists and TZD-like drugs for the treatment of diabetes. Further investigation of the role that diabetes medications play in mechanisms of insulin resistance and oxidative metabolism may provide insight into diabetes treatment.
